# Osteopenia and osteoporosis associated with hyperprolactinemic antipsychotics

**DOI:** 10.1192/j.eurpsy.2023.814

**Published:** 2023-07-19

**Authors:** A. L. Montejo, B. Buch, Y. Santana, J. Matías, J. Matias-Polo, T. Martín-Pinto, C. Matos, B. Cortés, R. de Alarcón, C. Fombellida, J. M. Acosta

**Affiliations:** 1 Psychiatry, University of Salamanca; 2 Instituto de Investigación Biomédica de Salamanca (IBSAL), IBSAL; 3 Psychiatry, University Hospital, Salamanca, Spain

## Abstract

**Introduction:**

The main role of prolactin is associated mainly with lactogenesis but additionally it participates in several endocrinological and metabolic processes. The prolactin level may be increased with some antipsychotics such as risperidone, paliperidone, and amisulpride increasing the risk of Bone Mineral Mass (BMM) decrease leading to osteopenia and osteoporosis.

**Objectives:**

To determine the loss of BMM associated with antipsychotic-related iatrogenic hyperprolactinemia (iHPRL) in a sample of patients suffering of chronic psychotic mental disorder and treated with antipsychotics at least for one year.

**Methods:**

A cross-sectional observational and epidemiological study in a sample of 140 patients (males 56.9%; females 43.1%; mean age 48 years), receiving antipsychotics was carried out. After giving informed consent, personal data, prolactin level, antipsychotic use and lifestyle were collected. An evaluation of BMM with a central DEXA Scan was performed. The bone mineral density considering the subject´s age and the peak bone mass in the neck of the femur, hip and in the lumbar vertebrae (L1-L4) was obtained. Inclusion criteria: presence of psychotic disorder, age between 18-65 years and treatment with an antipsychotic at least for one year. Statistical analysis was carried out using the statistical software SPSS version 26.0. A significance level α=0.05 was considered throughout the study.

**Results:**

45 out of 140 patients (32,13%) had some BMM lost (osteopenia). The prevalence of osteoporosis was 5.71% (n=8). The median prolactin level in the sample was 46.1 ng/dL ± 33.1. Patients with hyperprolactinaemia showed a higher frequency of osteopenia/osteoporosis (50% with mild iHPRL and 48% with moderate/severe iHPRL) than those with normal prolactin levels (25.7%). A strong and significant relationship between the presence of osteoporosis and the treatment with risperidone was found (p=0.007).

**Conclusions:**

Osteopenia and osteoporosis are associated with hyperprolactinemic antipsychotic. Risperidone was related with a significant increased osteoporosis risk. The rutinary and systematic control of the BMM is crucial in these patients to avoid progressive bone demineralization. Managing strategies should be individualized to avoid bone demineralization and to preserve physical health.

**Disclosure of Interest:**

None Declared

